# OA15 Missing the wood(-y induration) for the trees

**DOI:** 10.1093/rap/rkad070.015

**Published:** 2023-09-27

**Authors:** Fiona Rayner, Rebecca Batten

**Affiliations:** South Tyneside and Sunderland NHS Foundation Trust, Sunderland, United Kingdom; South Tyneside and Sunderland NHS Foundation Trust, Sunderland, United Kingdom

## Abstract

**Introduction:**

We describe a case that presented to the rheumatology department with classical signs of dermatomyositis including proximal muscle weakness, skin rashes and peripheral oedema. Effective treatment with corticosteroids uncovered important clinical signs, and along with results of investigations, this prompted us to revisit the diagnosis.

**Case description:**

A 67-year-old woman was referred to the rheumatology clinic with a 6-month history of proximal muscle weakness, peripheral oedema and facial rash in a malar distribution. She reported previously having a violaceous rash affecting her chest and back, which had subsequently resolved. The symptoms were associated with fatigue, fevers, breathlessness and a dry cough. She was admitted to the rheumatology ward directly from clinic with a working diagnosis of dermatomyositis and she was treated with 3 days of IV methylprednisolone. Initial bloods showed Hb 113, WBC 14.3, neutrophils 7.2, platelets 506, ESR 64, U&Es normal, CRP 32, CK 19, ANA 1/80 (homogenous pattern), negative ENA screen, negative myositis screen, normal complement, and hypergammaglobulinaemia at 24.2. Bilateral thigh MRI revealed features suggestive of diffuse myositis and muscle biopsy showed findings consistent with autoimmune inflammatory myopathy. Malignancy screen including CT chest, abdomen and pelvis, mammography and faecal immunochemical testing were negative. Pro-BNP was 560 and echocardiogram showed mild septal diastolic dysfunction. She was discharged on oral prednisolone with a plan to start rituximab and maintenance azathioprine. On review in clinic 2 weeks following discharge, her symptoms of weakness and peripheral oedema were much improved. With the oedema better, it was now evident that she had skin thickening on the forearms and induration of the veins on raising her arms (positive groove sign). On reviewing historic blood results, her eosinophil count had been significantly raised at 5.6 two months prior to presentation. The diagnosis was revised to eosinophilic fasciitis with myositis. The muscle biopsy was re-reviewed and a small section of skin that was present showed non-specific inflammation of a section of fascia but no eosinophilic infiltrate present. Following discussion in the regional MDT, treatment with pulsed IV cyclophosphamide and methylprednisolone was commenced with a plan to start methotrexate following this.

**Discussion:**

Eosinophilic fasciitis (EF) is a rare disease of unknown aetiology characterised by thickening of the skin on the limbs or trunk, which was first described in 1974. It can mimic scleroderma, although the skin of the fingers and feet are usually spared in EF. The skin changes are often described as woody induration, which can also be present in the later stages of scleroderma. The typical “groove sign”, when a furrow is present along the course of veins, is accentuated with elevating the affected limb. This is due to loss of perivenular fat and inward skin tethering. There can be a peau d’orange appearance and cobblestoning in the affected areas. The skin changes are usually bilateral and can be associated with oedema. Eosinophilia is present in 58-85% of cases and is commoner at the onset of disease.

Other features that can be present in EF include inflammatory arthritis, joint contractures, myalgias, myositis, neuropathies and rarely visceral involvement. EF can be associated with haematological disorders such as aplastic anaemia, myeloproliferative disorders, lymphoma and multiple myeloma. Diagnosis of EF is usually confirmed with a skin biopsy. In early disease this shows oedema and a lymphocytic infiltrate with plasma cells and eosinophils. Later in the disease there is collagen thickening and sclerosis. There have been case reports of EF with myositis showing an inflammatory infiltrate in the muscles similar to the findings in inflammatory myositis. More recently a multicentre retrospective case series examined patients with idiopathic eosinophilic myositis (IEM) and EF and proposed a continuum of disease from eosinophilic myositis (focal or diffuse) through to eosinophilic myofasciitis and finally eosinophilic fasciitis, with varying levels of muscle and skin involvement.

**Key learning points:**

If the diagnosis doesn’t fit, review old notes and tests

Our patient presented with classic features of dermatomyositis (skin rash, weakness, myalgia) but a normal CK and negative myositis screen prompted consideration of other diagnoses. Reviewing old blood results was crucial in this case, as eosinophilia is sometimes only present in EF at the onset of disease. Case reports of muscle involvement in EF suggest that some patients may fall into a spectrum of disease between EF and inflammatory myositis. Our patient most likely fits with a diagnosis of eosinophilic myofasciitis which is characterised by subjective muscle weakness, fasciitis and hypereosinophilia.

Use investigations to differentiate EF from myositis, overlap syndromes and scleroderma mimics

Patients with EF are generally ANA negative and those with a myositis overlap are more likely to have normal CK compared with patients presenting with pure inflammatory myositis. It is common to see a hypergammaglobulinaemia in EF. An MRI of the affected area and crucially a full thickness skin biopsy and muscle biopsy can help to determine where on the spectrum a patient sits. There are many other rare diseases that can present with skin thickening like EF such as systemic sclerosis, localised scleroderma, scleredema, scleromyxedema and nephrogenic systemic fibrosis, so a skin biopsy is vital to help with the diagnosis.

EF is steroid responsive

If caught early in the oedematous phase, EF responds well to steroids and 50% of patients can achieve remission on steroids alone. An initial dose of prednisolone 1mg/kg is recommended. Steroid sparing agents should be used with most evidence supporting methotrexate. Physiotherapy should be employed to prevent and treat contractures. These sometimes require surgical release or fasciectomy.

